# A Randomized Exploratory Study Comparing the Success Rate of Ultrasound Guided Central Venous Catheterization With a Curved Tip Needle Versus a Conventional Straight Needle in Adult Patients

**DOI:** 10.7759/cureus.103137

**Published:** 2026-02-06

**Authors:** Mridul Dhar, Vijay Adabala, Anirban B Adhikary, Pulkit Johar, Osharani Rathod, Azhar Khan

**Affiliations:** 1 Anaesthesiology, All India Institute of Medical Sciences, Rishikesh, Rishikesh, IND; 2 Anaesthesiology, All India Institute of Medical Sciences, Rae Bareli, Rae Bareli, IND

**Keywords:** catheterization, central venous catheterization, central venous catheters, jugular vein, ultrasonography

## Abstract

Introduction: Central venous catheter insertion is routinely performed in adults with high success rates under ultrasound guidance, but guidewire advancement can be difficult in patients with small-caliber veins, particularly in neonates and infants. This randomized exploratory study in adult patients evaluates whether a curved-tip needle, conceptually designed for use in smaller veins, performs at least as well as a standard needle in internal jugular vein (IJV) cannulation.

Methods: In this prospective, randomized exploratory study, adult patients requiring ultrasound-guided IJV cannulation for any indication were included. Forty participants were randomized into two groups: the curved group (CG), using a curved tip needle (10°), and the standard group (SG), using a conventional straight needle. The primary outcome was the first pass success rate. Secondary objectives were overall success rate, number of attempts, needle visualization, and complications.

Results: Results were similar in both the CG and SG in terms of first pass success rate (95%), overall success rate (100%), and number of attempts. Needle visualization under ultrasound was similar in both groups. No complications were noted in either group.

Conclusion: Use of a curved tip needle for IJV catheterization in adults showed similar success rates and needle visualization compared with a conventional straight needle, with no added complications. Further research may explore its utility in non-operative settings, in smaller caliber veins, and at alternative central venous sites.

## Introduction

Central venous catheters (CVCs) are routinely inserted for various perioperative indications as well as in critically ill patients. The CVC insertion process in adults is fairly simple and standardized, but it often becomes challenging in younger patients, in whom the caliber of vessels is significantly smaller and equipment- and guidewire-related issues make the insertion process unpredictable and often tedious, even with the use of ultrasound (US) [1,2]. One of the key steps in CVC insertion is threading the guidewire through the insertion needle. This step is especially difficult in neonates and infants and at insertion sites other than the internal jugular vein (IJV). Although most commercially available pediatric guidewires have a J tip to avoid injury to the vessel wall, this feature often becomes a hindrance to smooth advancement of the guidewire further into the vein because the guidewire may abut the vessel walls [3,4]. Using a peripheral intravenous cannula in place of a conventional CVC insertion needle has also been used as an alternative to insert the guidewire, with some anecdotal success, especially in femoral vein catheterization, due to its better flexibility and tendency to remain aligned with the vessel wall [5].

Pre-bent needles have been studied in previous research during neuraxial procedures, such as epidural and spinal needle placement [6]. A similar concept can be applied to the tip of the central line insertion needle, with the intention of facilitating guidewire insertion. This approach may be particularly useful in smaller caliber vessels in pediatric patients. This exploratory study in adult patients investigates the hypothesis that a conventional CVC insertion needle, when bent at the tip by 10° before vessel entry, becomes more aligned with the vessel wall, thereby facilitating easier guidewire advancement.

The primary research question was whether a curved-tip needle is non-inferior to a straight needle for first-pass success and visualization in adult IJV cannulation, thereby justifying further evaluation in pediatric patients and more challenging venous access scenarios. The primary objective was to compare the first-pass success rate of a curved-tip central line needle with that of a conventional straight needle for central venous catheterization. Secondary objectives included comparison of ultrasonographic visualization of the central line needle, overall procedure success rate, number of attempts, and the incidence of complications between the two groups.

## Materials and methods

This was a single-centre, open-label, parallel-group randomized exploratory trial comparing a curved-tip versus a conventional straight needle for ultrasound-guided right internal jugular venous cannulation in adults. It was initiated after approval of the institutional ethics committee and registration of the study in the Clinical Trial Registry of India (CTRI/2022/07/044044). The study was conducted at the All India Institute of Medical Sciences, Rishikesh, India, between January 1, 2024, and August 12, 2024. Written informed consent was taken from all participants, along with permission to use the data for educational and research purposes. The inclusion criteria were adult patients of either gender, between 18 and 60 years, requiring IJV central line insertion for any indication. The right-sided IJV was selected for uniformity of procedure. Patients with uncorrected coagulopathy, infection at the site of insertion, pre-existing thrombus in the IJV, sepsis, or hemodynamic instability were excluded from the study. The setting of the study was the operating theatre or wards of various medical and surgical specialties of a tertiary care center. The conduct of the study and preparation of the article followed the Consolidated Standards of Reporting Trials guidelines and the principles of the Declaration of Helsinki (2013) and good clinical practice.

**Figure 1:**
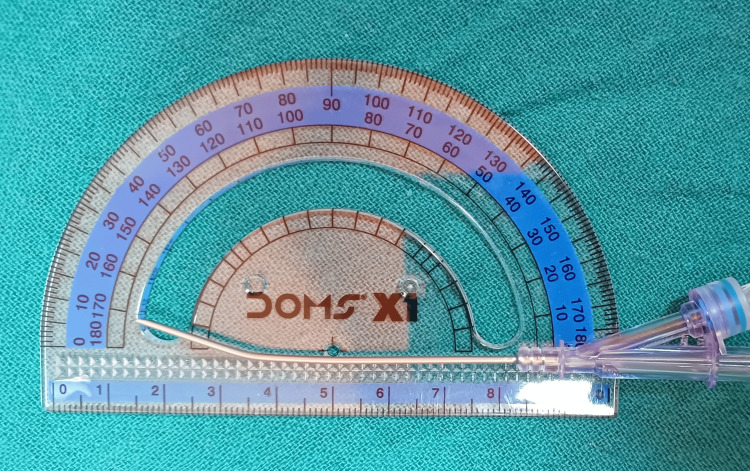
Use of a protractor to measure the angle of needle tip bending

**Figure 2:**
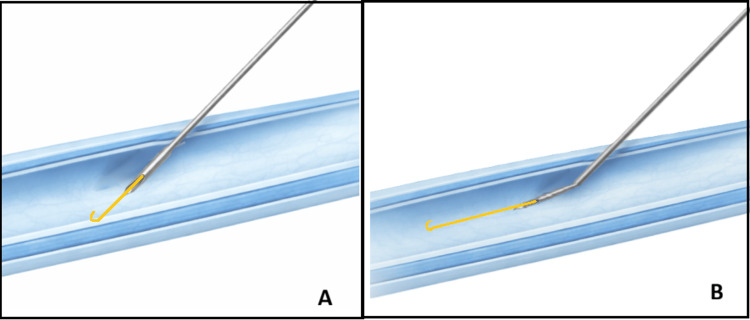
Illustration (not to scale) depicting guidewire path with (a) standard straight needle hitting the posterior vessel wall and (b) curved tip needle, aligned with the vessel wall

Randomization was done using computer-generated blocks of six (studyrandomizer.com) with an allocation ratio of one. The numbers were sealed in opaque envelopes to conceal the allocation sequence, which was prepared by the primary investigator. This was an open-label study. After written informed consent, patients were taken to the operating theatre, the clinical ward, or procedure area with standard monitoring (non-invasive blood pressure, pulse oximetry, and electrocardiogram), and functional intravenous access was secured or ensured. If a surgical case, general anesthesia was induced before central line insertion as per the requirements of the case and the indication for central venous access. The procedure assistant or nurse opened the envelope to reveal the allocated group. The enrolled patients were randomized into the curved group (CG), which included the curved tip needle, or the standard group (SG), which included the conventional straight needle. The intervention in the CG was a curved needle made by creating a gentle bend of 10°, two cm proximal to the tip of the needle (Figure 1). The control in the SG was the conventional needle without any modification. For the CG, the primary researcher bent the needle in all cases using artery forceps guided by a protractor in a sterile manner once the group allocation was revealed. The bend was created in a graded manner while aligning it against a protractor. The bevel of the needle was kept facing up relative to the plane of bending (Figure 1). Smooth guidewire movement in the needle was confirmed before insertion in the patient. During early feasibility testing in non-study patients, a 15° bend occasionally impeded wire passage, leading to protocol modification to 10°; subsequently, no resistance was observed. B Braun Certofix® catheters (7 Fr double or triple lumen as per indication) with an 18G, 7-cm Y insertion needle with a thin wall were used. The concept of the bend, as mentioned earlier, was to avoid the guidewire hitting the posterior vessel wall at the time of advancement through the insertion needle (Figure 2).

For the CVC insertion procedure, sterile preparation of the neck at the IJV insertion site was done. A linear US transducer was used (Edge-II, Fujifilm Sonosite Inc., Bothell, Washington). After the initial anatomical scan, 1-2 mL of local anesthesia, lignocaine (2%) with adrenaline (1:2,00,000), was injected at the needle insertion site if the patient was awake. Under US guidance using the out-of-plane technique, the needle was inserted into the IJV until free flow of venous blood was observed. The guidewire was then inserted, and its position was confirmed by US. Finally, the CVC was railroaded over the guidewire and inserted to the appropriate length. Free flow of blood was confirmed in all ports on aspiration, and the catheter was secured in place with sutures. The procedure was performed by an anesthesiologist not part of the study team and who had a minimum experience of three years as an anesthesiologist or experience of at least 50 central venous cannulations. A 10-degree head-down position was applied to the table in all patients during needle entry.

First pass success rate was the primary outcome (defined as successful guidewire placement and subsequent catheter advancement into the IJV with a single skin puncture, with one needle redirection within the same skin puncture to achieve adequate blood aspiration). Secondary outcomes were overall success rate (success within three attempts of the index procedure by the same operator at the same site), number of attempts taken, US visualization of the needle, and mechanical complications, if any were noted. A 10-point scale was used for visualization of the CVC needle during the entire needle insertion trajectory, till guidewire insertion (the scale was anchored wherein one equals 0%-10% and 10 equals 90%-100% of needle visualization) [7]. Other data, such as demographic data including age, gender, weight, height, and type of surgery, were also recorded. In case of failure of the procedure for IJV on the right side (defined as three or more attempts at needle insertion), the alternative side or sites were attempted, or the procedure was abandoned if feasible for surgery or disease with alternate peripheral access. The outcomes were assessed by a member of the research team, except for the US needle visualization, which was conveyed by the anesthesiologist performing the procedure or operator (after explaining the scoring to him or her).

Sample size and interim analysis: In the absence of prior studies or pilot data for expected success rates with a curved tip needle, the study was planned as an exploratory comparison. A pragmatic sample size of 30 participants per group was selected to allow estimation of group-wise success proportions and to generate preliminary effect size estimates for future confirmatory studies. This sample size is consistent with recommendations for pilot studies comparing proportions. A pre-planned interim analysis (no formal stopping rules for efficacy were applied) was done with 20 patients in each arm. As no clinically meaningful difference or trend was noted between the groups, the study was concluded at this stage. The present report reflects results from this interim analysis. A priori limitation was that the study may not be adequately powered to detect small or moderate differences in success rate or rare complications.

Data were recorded in an Excel 2019 spreadsheet (Microsoft Corporation, Redmond, Washington). Instat version 3.05 (Graphpad Inc., Boston, Massachusetts) was used for data analysis. Numerical data were expressed as mean (SD) or median (quartiles), and categorical data in percentages. Comparative analysis of the first pass success rate, overall success rate, and complication rate was done using either the chi-square test or Fisher’s exact test with effect estimates and 95% confidence interval (CI). The number of attempts and needle visualization score were compared with either an unpaired t-test or the Wilcoxon test based on the normality of the data. Intention-to-treat analysis was done for all outcomes. The p-value was considered significant at less than 0.05.

## Results

**Figure 3:**
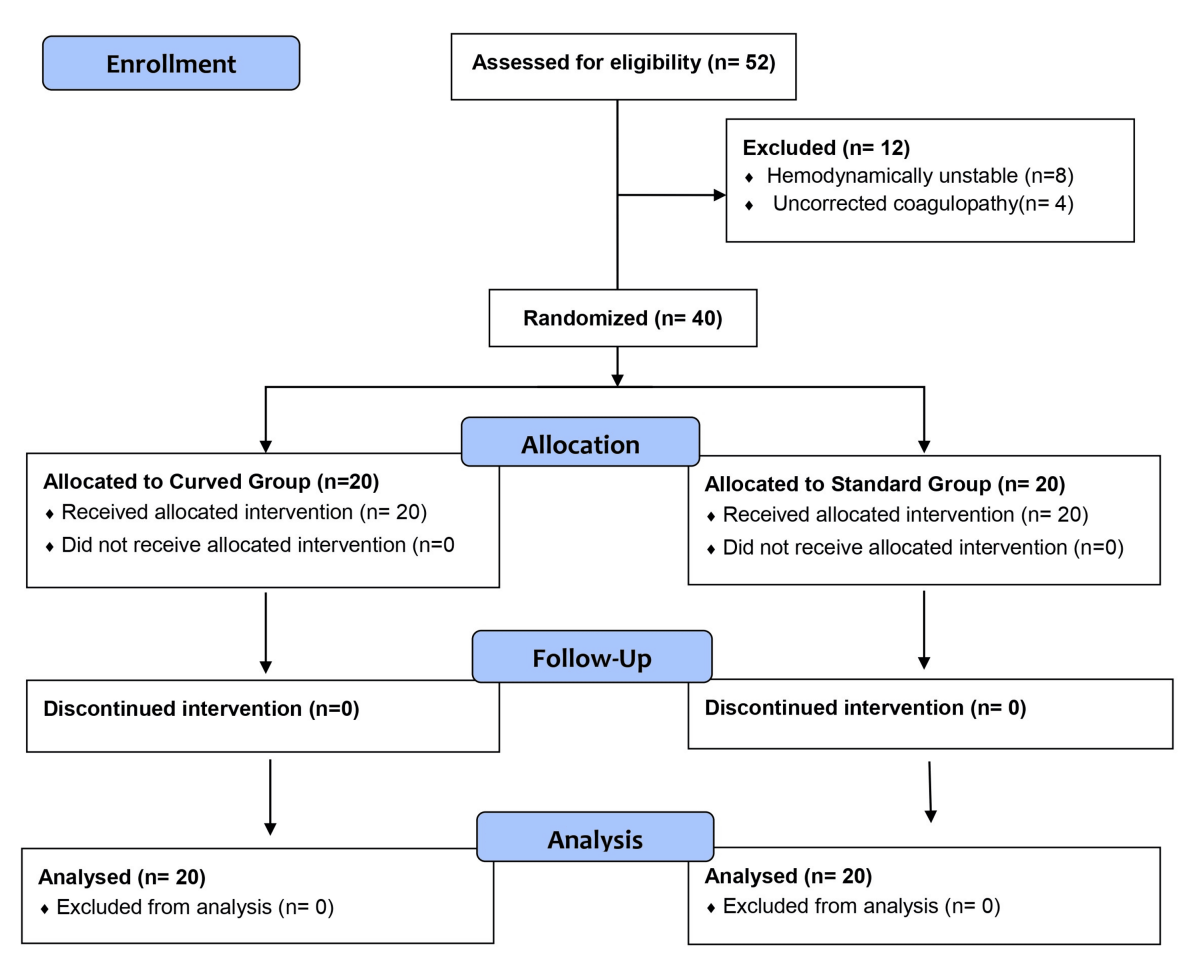
Consolidated Standards of Reporting Trials (CONSORT) flow diagram for participant enrolment

A total of 52 patients were considered for enrolment initially, of whom 40 were randomized at the time of the interim analysis (Figure 3). There were no protocol deviations in either group. Both groups were comparable in terms of demographic variables and baseline characteristics. All patients were surgical patients, with the central line secured after GA. The majority of patients in both groups were undergoing abdominal surgery, and there was no significant difference in the type of surgery, with both groups being well balanced in terms of surgical mix and anesthetic conditions (Table 1). The procedures were performed by three senior resident doctors with equal experience.

**Table 1:** Demographic and surgical data Data expressed as mean ± standard deviation or frequency (%). CG: curved group, SG: standard group, t: unpaired Student t-test, χ²: chi-square test, df: degree of freedom.

Parameter	CG (n= 20)		SG (n= 20)	Test value	P-value
Age (years)	44.65 ± 16.03		47.7 ± 10.34	t = 0.715, df = 38	0.479
Height (cm)	160.4 ± 7.48		156 ± 9.34	t = 1.644, df = 38	0.108
Weight (kg)	59.75 ± 10.23		55.05 ± 7.92	t = 1.6425, df = 38	0.112
Gender					
Male	11 (55%)		11 (55%)	χ² = 0, df = 1	1
Female	9 (45%)		9 (45%)		
Type of surgery					
Abdominal	14 (70%)		13 (65%)	χ² = 7.37, df = 5	0.195
Orthopedic	3 (15%)		1 (5%)		
Urological	1 (5%)		2 (10%)		
Head and neck	0 (%)		3 (15%)		
Thoracic	0 (%)		1 (5%)		
Extremity surgery	2 (10%)		0 (%)		

The primary outcome, first-pass success rate, was identical in both groups (95% vs 95%; risk difference 0%, with a 95% CI (−14% to +14%), reflecting uncertainty due to the small sample size (Table 2).

**Table 2:** Comparison of success rate and needle visualization Data expressed as median (Quartiles1 and 3) or frequency (%). CG: curved group, SG: standard group, FET: Fisher’s exact test, U: Mann–Whitney U-test, CI: confidence interval, RD: risk difference, MD: median difference.

Parameter	CG (n= 20)	SG (n= 20)	Test value	P value	Effect size (95% CI)
First pass success rate	19 (95%)	19 (95%)	FET	1.000	RD: 0% (−14% to +14%)
Needle visualization with the ultrasound (1-10)	9 (8.5,9.5)	9 (8,9.5)	U = 169	0.406	MD: 0 (−1 to +1)
Overall success rate	20 (100%)	20 (100%)	-	-	-
Number of attempts	1(1,1)	1(1,1)	U = 199.5	0.999	MD: 0 (0 to 0)
Complications	Nil	Nil	-	-	-

**Figure 4:**
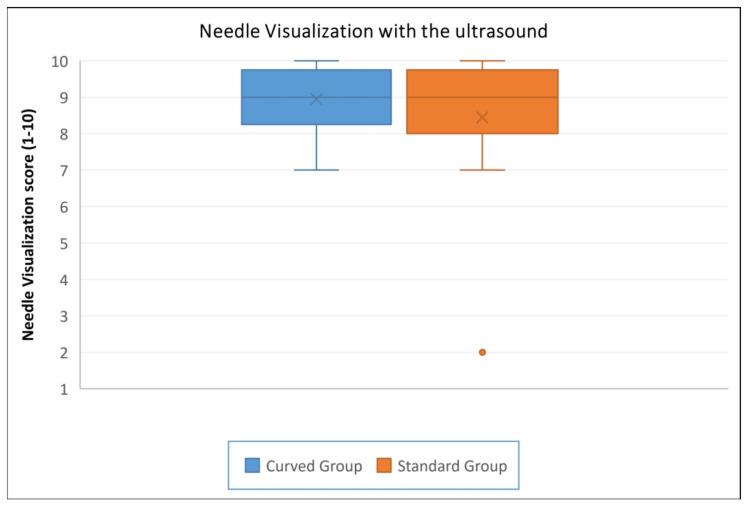
Box and Whisker plot of needle visualization score with ultrasound

Secondary outcomes showed that median needle US visualization scores were 9 (8.5, 9.5) in the CG and 9 (8, 9.5) in the SG, with a median difference of 0 and a 95% CI (−1 to +1), indicating no meaningful difference in ultrasound needle visualization (Table 2, Figure 4). Overall cannulation success was 100% in both groups (20/20 versus 20/20). As there were no failures in either group, no statistical comparison was performed. The median number of attempts was 1 (1, 1) in both the CG and SG, with a median difference of 0 and a 95% CI (0 to 0), indicating no difference in the number of attempts required (Table 2). No complications were observed in either group.

## Discussion

In this randomized exploratory trial of adult ultrasound-guided right IJV cannulation, a 10° curved-tip needle had similar first-pass and overall success rates, ultrasound visualization scores, and complication rates compared to a standard straight needle.

Various alternative techniques have been used to improve the success rate of US-guided CVC insertion in both children and adults. These include the use of needle guides and tracking, as well as technologies such as augmented reality and 3D or 4D ultrasound, among others [8,9]. The key or rate-limiting step in the procedure is guidewire advancement into the vessel, which becomes more critical in younger patients. Although the problem statement is more relevant to pediatric patients, our adult data suggest that a curved-tip needle is at least feasible and not obviously inferior. Thus, further targeted evaluation is needed before extrapolating these findings to infants or neonates or to alternative sites [10].

Previous studies have reported failure to advance the guidewire while using a straight needle even after adequate backflow [11]. In the present study, the final angle used in the CG was 10°. This angle produced a gentle curve that did not obstruct guidewire movement within the needle. Studies have also analyzed newer techniques, such as the “wire in needle” technique, to increase the likelihood of successful guidewire entry [12].

In the present study, the first-pass success rate, overall success rate, and number of attempts were similar when using either a straight or curved needle. Slight manipulation of the needle position and the gentle Trendelenburg position allowed easy passage of the guidewire, even when slight resistance was encountered during the initial attempt in both groups. The IJV has a relatively straighter course compared to other sites of central line insertion. In addition, the reported success rate of IJV cannulation in the literature is already high with a conventional insertion needle, with or without the use of US [13,14]. This, along with experienced operators, leaves little room for improvement. Hence, any ergonomic benefit of a curved needle may only become apparent in more challenging scenarios, such as small-caliber veins, less experienced operators, and alternative sites such as the femoral or subclavian vein [15]. The curved tip design may still confer benefits in adequately powered samples, particularly for subtle ergonomic advantages and fewer posterior wall punctures.

Needle tip visualization by ultrasound was similar in both groups, and the bent tip did not hamper US visualization. In the current study, the out-of-plane approach was used. Previous studies have advocated combined or long-axis approaches for improved needle visualization to avoid posterior wall puncture, as well as the use of marked needle tips [16,17]. However, the in-plane or long-axis approach may not always be feasible due to logistical and space constraints, especially in neonates. In this context, a curved-tip needle may represent a safer option when using the out-of-plane approach, as it allows a smaller angle of initial skin puncture rather than tilting the needle downward after vessel puncture to remain aligned with the vessel wall. Newer biplane or three-dimensional visualization compatible machines are now available to improve safety and first-pass success rates [9,13]. A combination of innovative US approaches and improved needle ergonomics may reduce guidewire failures.

No complications were observed in either group. Mechanical complications during IJV cannulation are infrequent, particularly when procedures are performed by experienced operators using US guidance. This may explain the absence of complications in both groups, although the sample size was small and not powered to detect differences in rare complications [18]. Needle geometry and ergonomics generally have a greater impact on first-pass success rates than on complication rates [16].

The strength of this study was that it was exploratory in nature, with the aim of establishing parity or feasibility of using a curved needle for central venous catheter insertion in adults. The limitations were as follows. The study included a single interim analysis, after which recruitment was stopped. As no formal adjustment for interim analysis was undertaken, the study may have reduced power compared to the originally planned sample size, with a corresponding increase in the risk of a Type II error. The single-center design and restriction to a single site, right IJV, and a specific setting, elective surgical patients under GA with experienced anesthesiologists, limit generalizability to emergency, critical care, pediatric, and non-IJV settings. The open-label design may also have introduced bias in subjective scoring. Other outcomes relevant to needle ergonomics, such as procedure time, posterior wall puncture, and operator satisfaction, were not assessed in the current study.

## Conclusions

Using a curved tip needle for central venous catheterization in adults provides a similar success rate and US needle visualization compared with straight needles, without additional complications. In adult right IJV cannulation under ultrasound, a manually curved 10° needle appears feasible and non-inferior to a standard needle; however, routine adoption cannot be recommended based on these data alone. Future directions include multicenter pediatric trials focusing on neonates and infants with prespecified endpoints of guidewire failure rate, time to cannulation, and complications, as well as trials targeting more challenging sites, such as the femoral and subclavian veins, and settings with lower baseline success, including emergency departments and low-resource settings.
